# Evaluation of the prognostic value of GDF-15, ABC-AF-bleeding score and ABC-AF-death score in patients with atrial fibrillation across different geographical areas

**DOI:** 10.1136/openhrt-2020-001471

**Published:** 2021-03-19

**Authors:** Tymon Pol, Ziad Hijazi, Johan Lindbäck, John H Alexander, M Cecilia Bahit, Raffaele De Caterina, JW Eikelboom, Michael D Ezekowitz, Bernard J Gersh, Christopher B Granger, Elaine M Hylek, Renato Lopes, Agneta Siegbahn, Lars Wallentin

**Affiliations:** 1 Department of Medical Sciences, Cardiology, Uppsala University, Uppsala, Sweden; 2 Uppsala Clinical Research Center, Uppsala University, Uppsala, Sweden; 3 Duke Clinical Research Institute, Duke Health, Durham, North Carolina, USA; 4 INECO Neurociencias Oroño, Fundación INECO, Rosario, Argentina; 5 Gabriele d'Annunzio University, Chieti, Italy; 6 Population Health Res Inst, Hamilton, Ontario, Canada; 7 Thomas Jefferson Medical Coll and the Heart Ctr, Wynnewood, Pennsylvania, USA; 8 Mayo Clinic College of Medicine, Rochester, Minnesota, USA; 9 Boston Univ Medical Ctr, Boston, Massachusetts, USA; 10 Department of Medical Sciences, Clinical Chemistry, Uppsala University, Uppsala, Sweden

**Keywords:** atrial fibrillation, biomarkers, global health

## Abstract

**Objectives:**

Growth differentiation factor 15 (GDF-15) is a biomarker independently associated with bleeding and death in anticoagulated patients with atrial fibrillation (AF). GDF-15 is also used as one component in the more precise biomarker-based ABC (age, biomarkers, clinical history)-AF-bleeding and ABC-AF-death risk scores. Data from large trials indicate a geographic variability in regard to overall outcomes, including bleeding and mortality risk. Our aim was to assess the consistency of the association between GDF-15, ABC-AF-bleeding score and ABC-AF-death score, with major bleeding and death, across world geographic regions.

**Methods:**

Data were available from 14 767 patients with AF from the Apixaban for Reduction in Stroke and Other Thromboembolic Events in Atrial Fibrillation (ARISTOTLE) trial and 8651 patients with AF from the Randomized Evaluation of Long-Term Anticoagulation Therapy (RE-LY) trial in this cohort study. GDF-15 was analysed from plasma samples obtained at randomisation. The geographical consistency of the associations between outcomes and GDF-15, ABC-AF-bleeding score and ABC-AF-death scores were assessed by Cox-regression models including interactions with predefined geographical region.

**Results:**

GDF-15 and the ABC-AF-bleeding score were associated with major bleeding in both trials across regions (p<0.0001). Similarly, GDF-15 and the ABC-AF-death score were associated with all-cause mortality in both trials across regions (p<0.0001). Overall, the association between GDF-15, the ABC-AF-bleeding score and ABC-AF-death risk score with major bleeding and death was consistent across regions in both ARISTOTLE and the RE-LY trial cohorts. The ABC-AF-bleeding and ABC-AF-death risk scores were consistent regarding discriminative ability when comparing geographic regions in both trial cohorts. The C-indices ranged from 0.649 to 0.760 for the ABC-AF-bleeding and from 0.677 to 0.806 for the ABC-AF-death score by different geographic regions.

**Conclusions:**

In patients with AF on anticoagulation, GDF-15 and the biomarker-based ABC-AF-bleeding and ABC-AF-death risk scores are consistently associated with respectively increased risk of major bleeding and death and have similar prognostic value across world geographic regions.

**Trial registration number:**

ClinicalTrials.gov Registry NCT00412984 and NCT00262600.

**Key questions**

**What is already known about this subject?**

- Growth differentiation factor 15 (GDF-15) and the biomarker-based ABC-AF-bleeding and ABC-AF-death risk scores are independently associated with bleeding and death in anticoagulated patients with atrial fibrillation (AF). Their prognostic value across geographical regions has however not been evaluated previously.

**What does this study add?**

- This study shows that GDF-15 and the ABC-AF-bleeding and ABC-AF-death risk scores have similar prognostic value across world geographic regions.

**How might this impact on clinical practice?**

- The findings from this study add novel information regarding the consistency of GDF-15 and the ABC-AF-risk scores in AF and strengthen their role as a risk refinement tool applicable for a wide international usage. Furthermore, the assessment of geographic variation might be helpful as part of a validation process when introducing a new biomarker or risk score intended for a wide use.

## Introduction

In patients with atrial fibrillation (AF) an individual risk assessment is recommended.^1^ Several biomarkers have recently been shown to add independent prognostic value regarding the risk of bleeding and death in AF.^2–5^ Growth differentiation factor 15 (GDF-15) is a marker of oxidative stress and inflammation and has been demonstrated to be strongly associated with major bleeding and death, but less with stroke, in patients with AF.^6 7^ These properties make GDF-15 an attractive candidate in the search for markers that facilitate the distinction between the risk of stroke against the risk of bleeding. GDF-15 has furthermore been incorporated into the two clinical risk scores in AF, the ABC-AF-bleeding and the ABC-AF-death scores (age, biomarkers and clinical history), providing improved risk assessment concerning these outcomes in AF and outperforming other risk scores in this scenario.^8 9^ However, recent data from large Non-Vitamin K Antagonist Oral Anticoagulants) trials have indicated a geographic variability of cardiovascular outcomes, including bleeding risk and mortality risk, even after adjustment for clinical factors and biomarkers.^10–14^ At present, it is unknown whether the risks associated with GDF-15 for major bleeding and death are similar across geographic regions, as well as the performance of the ABC-AF-bleeding and ABC-AF-death scores, in which GDF-15 is incorporated. Therefore, in this study, we aimed to evaluate GDF-15 and the ABC-AF-bleeding and ABC-AF-death scores across geographical regions using data from both the Apixaban for Reduction in Stroke and Other Thromboembolic Events in Atrial Fibrillation (ARISTOTLE) trial and the Randomized Evaluation of Long-Term Anticoagulation Therapy (RE-LY) trial.

## Methods

### Study population and trial design

The details and outcomes of the ARISTOTLE and RE-LY trials have been described and published previously.^15–18^ The ARISTOTLE trial randomised 18 201 patients from 39 countries with AF and at least one additional risk factor for stroke or systemic embolism to either warfarin or apixaban. The participating countries were categorised into prespecified regions: Asia/Pacific, Europe, Latin America and North America (online supplemental figure 1). This biomarker substudy used data from 14 949 patients with plasma collected at randomisation, out of which 14 688 had all biomarkers available, with a median follow-up of 1.8 (IQR: 1.3, 2.3) years. In the RE-LY trial, 18 113 patients with AF were randomly assigned to dabigatran or warfarin. In total, patients from 44 countries were included in this trial. These countries were divided according to geographic regions (Asia, Europe, Latin America, North America and other) (online supplemental figure 1). A total of 9369 patients had biomarker data available from samples collected at randomisation, out of which 8402 had all biomarkers available, with a median follow-up time of 2.0 (IQR: 1.7, 2.3) years.

10.1136/openhrt-2020-001471.supp1 Supplementary data

In both trials, all-cause mortality was a prespecified outcome and major bleeding was the primary safety outcome.^15 18^ Major bleeding was in both trials defined according to the criteria of the International Society on Thrombosis and Haemostasis.^15 18^ Blinded clinical events committees reviewed and centrally adjudicated these outcome events.

#### The ABC-AF-bleeding and ABC-AF-death scores

As previously described, the ABC-AF scores for prognostication of outcomes were developed with the objective to include a minimal number of the strongest markers among the candidate variables: A=Age, B=Biomarkers and C=Clinical factors.^8 9^The ABC-AF- bleeding score therefore consists of age, the levels of haemoglobin, high-sensitive cardiac troponin T (cTnT-hs) and GDF-15 and finally previous bleeding as the only necessary clinical factor. The ABC-AF-death score was developed in a similar way and incorporates age, the biomarkers N-terminal pro B-type natriuretic peptide (NT-proBNP), cTnT-hs, GDF-15 and a clinical history of heart failure.

### Laboratory methods

Venous blood samples were obtained at randomisation from enrolled patients in both trials. The samples were centrifuged and plasma stored in aliquots and subsequently transferred to the Uppsala Clinical Research Centre (UCR) laboratory, Uppsala, Sweden, for storage at −70°C and central analyses.^15 18^ GDF-15 was analysed with the Elecsys GDF-15 precommercial assay kit P03 (Roche Diagnostics, Mannheim, Germany). The assay is reported to have an interassay coefficient of variation of 2.3% at 100 ng/L and 1.8% at 17 200 ng/L; the intra-assay coefficient of variation was 0.8% at 1100 ng/L and 0.9% at 18 600 ng/L with a lower detection limit <10 ng/L. When intra-assay and interassay variabilities were combined, the coefficients of variation at our laboratory were 4.4% at 1500 ng/L and 4.5% at 5900 ng/L.^6^ The details for the analyses of the other biomarkers in the ABC-AF-bleeding and ABC-AF-death scores have previously been published, in short, cardiac troponin-T (cTnT-hs) and NT-proBNP levels were analysed with high-sensitivity immunoassays on the Cobas Analytics e601 (Roche Diagnostics, Mannheim, Germany).^8 9^ All analyses were done according to the instructions of the manufacturer.

Data collected in the ARISTOTLE and RE-LY trials (case report form, outcomes, laboratory samples and analyses) were standardised and congruent in both individual trials for all participating. GDF-15 analyses were in both trials analysed centrally in the UCR laboratory. Specific information regarding data collection and handling can be found in the original trial publications.^15–18^

### Statistical analysis

To assess the consistency over geographic regions regarding the association between time to major bleeding (or time to death) and GDF-15, a Cox-regression model including GDF-15, geographic region and their interaction was fitted. To assess the consistency of the ABC-AF scores, a corresponding model was fitted including the ABC-AF-bleeding (ABC-AF-death) score, here represented by the estimated 1-year risk for major bleeding (death), instead of GDF-15. In all models, GDF-15 and the ABC-AF scores were log-transformed (natural logarithm) and represented as restricted cubic splines with four knots placed at the 5th, 35th, 65th and 95th sample percentiles of the corresponding variable. The interaction term only included the linear part of GDF-15 or the ABC-AF score, respectively. Thus, the test of the hypothesis of no interaction between geographic region and GDF-15 (or the ABC-AF score) is the test for the regression coefficient of region×ln(GDF-15) (or region×the ABC-AF score) being zero in the model.

The associations are illustrated graphically by plotting the estimated relative hazard, with corresponding 95% confidence bands. For the models including GDF-15, an arbitrary reference was set at a GDF-15 value of 1500 ng/L for someone in Europe. For the ABC-AF scores, the reference point was set to an arbitrary value of 2% 1-year risk, also for someone in Europe. The horizontal range of the curves approximately represents the range of GDF-15 (ABC-AF score) after removal of the 10 lowest and 10 highest values.

Harrell’s c-index was used to assess the discriminative ability of GDF-15 and the ABC-AF scores, both overall and within regions.

All analyses were done in R, V.3.5.2,^19^ using the rms^20^ add-on package.

## Results

### Demographic data

In total, 14 688 patients from the biomarker cohort of the ARISTOTLE trial as well as 8402 patients from the RE-LY trial had all biomarker concentrations available at randomisation. The number of patients and events in each trial as well as baseline demographics and biomarker levels according to the prespecified geographic regions of both trials are shown in tables 1 and 2. All participating countries in both trial cohorts are shown in online supplemental figure 1. The median age in the ARISTOTLE trial cohort was 70 (IQR: 63, 76) years and 64% were men, while in the RE-LY cohort, the median age was 72 (IQR: 67, 77) years with the same proportion of men, 64%. The largest proportion of patients was from Europe in both trial cohorts, 40% in ARISTOTLE and 54% in RE-LY.

**Table 1:** Baseline characteristics of the 14 949 patients in the ARISTOTLE cohort according to study region

	Asia/Pacific N=2389	Europe N=6027	Latin America N=2959	North America N=3574
Age, median (Q1–Q3)	69.0 (61.0–75.0)	69.0 (62.0–75.0)	70.0 (64.0–77.0)	72.0 (65.0–78.0)
Female	35.7% (853)	37.1% (2235)	38.0% (1125)	31.0% (1109)
BMI, median (Q1–Q3)	25.1 (22.6–28.0) [7]	29.1 (26.1–32.8) [21]	28.7 (25.4–32.6) [24]	30.2 (26.5–35.0) [19]
Systolic blood pressure, median (Q1–Q3)	130.0 (120.0–140.0) [8]	133.0 (123.0–143.0) [12]	130.0 (120.0–140.0) [2]	128.0 (117.0–138.0) [10]
Diabetes	24.7% (589)	22.7% (1370)	20.1% (595)	31.7% (1133)
Hypertension	81.0% (1936)	89.8% (5414)	88.8% (2629)	86.9% (3105)
Smoker	8.4% (200) [1]	9.9% (593) [9]	5.6% (166) [4]	7.2% (258) [0]
Permanent or persistent AF	89.6% (2141) [0]	80.8% (4867) [1]	91.5% (2706) [1]	83.2% (2973) [1]
Stroke/TIA	26.7% (637)	19.0% (1148)	15.3% (454)	15.8% (565)
Prior bleeding	15.8% (377)	12.1% (732)	13.2% (392)	26.1% (932)
Prior anaemia	3.8% (90) [0]	4.9% (298) [3]	3.5% (104) [6]	14.4% (514) [2]
Congestive heart failure	24.9% (596)	40.8% (2458)	33.4% (989)	16.8% (601)
Myocardial infarction	8.2% (196) [0]	15.0% (906) [0]	9.5% (280) [1]	15.2% (542) [0]
Peripheral arterial disease	2.5% (59) [0]	5.3% (317) [0]	3.7% (109) [1]	6.8% (244) [0]
CKD-EPI (mL/min/1.73 m^2^), median (Q1–Q3)	59.0 (47.2–72.8) [1]	56.5 (46.4–67.8) [7]	57.0 (45.5–70.5) [0]	52.4 (42.1–63.2) [0]
GDF-15 (ng/L), median (Q1–Q3)	1476.5 (1049.8–2167.5) [29]	1263.0 (919.5–1865.0) [88]	1362.0 (973.0–2012.0) [34]	1559.0 (1074.5–2297.5) [31]
Haemoglobin (g/L), median (Q1–Q3)	141 (130–153) [13]	143 (133–153) [13]	144 (134–155) [26]	141 (130–150) [18]
NT-proBNP (ng/L), median (Q1–Q3)	725.0 (377.8–1220.2) [13]	695.0 (339.2–1244.0) [57]	751.0 (397.0–1325.0) [10]	706.0 (370.0–1217.8) [8]
hs-cTnT (ng/L), median (Q1–Q3)	9.8 (7.0–14.7) [12]	10.8 (7.4–16.3) [53]	11.4 (7.9–17.6) [9]	11.7 (7.9–17.6) [9]
Outcomes				
Death	152 (6%)	330 (5%)	332 (11%)	245 (7%)
Major bleeding	130 (5%)	206 (3%)	138 (5%)	195 (5%)
Missing GDF-15	28 (1%)	88 (1%)	34 (1%)	31 (1%)

Number in brackets represents number of missings.

AF, atrial fibrillation; ARISTOTL, Apixaban for Reduction in Stroke and Other Thromboembolic Events in Atrial Fibrillation; BMI, body mass index; CKD-EPI, Chronic Kidney Disease Epidemiology Collaboration equation; GDF-15, growth differentiation factor 15; hs-cTnT, high-sensitive cardiac troponin T; NT-proBNP, N-terminal pro B-type natriuretic peptide; Q, quartile; TIA, transient ischaemic attack.

**Table 2:** Baseline characteristics of the 9369 patients in the RE-LY cohort according to study region

	Asia N=891	Europe N=5024	Latin America N=629	North America N=2082	Other N=743
Age, median (Q1–Q3)	69.0 (63.0–75.0)	72.0 (66.0–77.0)	73.0 (67.0–78.0)	74.0 (68.0–79.0)	74.0 (69.0–79.0)
Female	32.9% (293)	36.9% (1855)	40.1% (252)	34.1% (711)	40.0% (297)
BMI, median (Q1–Q3)	24.3 (22.0–27.0) [3]	28.1 (25.4–31.4) [1]	27.9 (25.3–31.6) [0]	29.0 (25.7–33.3) [3]	28.4 (25.3–31.6) [0]
Systolic blood pressure, median (Q1–Q3)	130.0 (120.0–140.0) [2]	132.0 (120.0–145.0) [8]	131.0 (120.0–144.0) [0]	128.0 (118.0–140.0) [2]	130.0 (120.0–143.8) [1]
Diabetes	25.3% (225)	20.4% (1024)	16.2% (102)	24.4% (509)	29.5% (219)
Hypertension	71.6% (638)	78.7% (3954)	82.0% (516)	81.4% (1694)	79.3% (589)
Smoker	8.4% (75)	8.6% (432)	4.8% (30)	6.3% (132)	7.0% (52)
Permanent or persistent AF	69.7% (621) [0]	71.3% (3580) [0]	87.3% (549) [0]	5.2% (1147) [4]	58.7% (436) [0]
Stroke/TIA	29.4% (262)	18.8% (944)	14.9% (94)	16.8% (349)	23.4% (174)
Anaemia	22.2% (198)	10.2% (513)	7.5% (47)	14.0% (291)	18.0% (134)
Congestive heart failure	34.9% (311) [0]	33.8% (1696) [0]	33.9% (213) [0]	16.0% (334) [1]	21.1% (157) [0]
Myocardial infarction	10.2% (91)	17.2% (864)	7.5% (47)	19.6% (408)	24.1% (179)
Peripheral arterial disease	0.4% (4) [0]	3.8% (193) [0]	2.2% (14) [0]	5.0% (105) [0]	3.9% (29) [1]
CKD-EPI (mL/min/1.73 m^2^), median (Q1–Q3)	70.7 (58.5–85.4) [0]	65.5 (54.5–76.9) [75]	57.9 (47.5–68.3) [0]	64.2 (53.3–76.4) [8]	63.1 (52.2–75.2) [8]
GDF-15 (ng/L), median (Q1–Q3)	1606.0 (1181.5–2407.5) [76]	1436.0 (1060.0–2067.0) [365]	1545.0 (1142.2–2081.2) [19]	1608.0 (1182.0–2332.0) [171]	1744.5 (1231.5–2605.8) [87]
Haemoglobin (g/L), median (Q1–Q3)	139 (127–151) [3]	144 (134–154) [103]	144 (134–155) [4]	141 (131–151) [29]	140 (128–150) [20]
NT-proBNP (ng/L), median (Q1–Q3)	807.0 (400.5–1576.5) [3]	837.0 (396.0–1477.0) [3]	819.0 (453.0–1410.0) [8]	748.0 (368.5–1354.0) [19]	854.0 (372.5–1406.5) [8]
hs-cTnT (ng/L), median (Q1–Q3)	10.8 (7.1–18.0) [39]	12.2 (7.7–19.5) [385]	12.9 (8.3–20.0) [21]	12.0 (7.6–19.0) [121]	13.8 (8.5–22.8) [84]
Outcomes					
Death	66 (7%)	341 (7%)	54 (9%)	104 (5%)	42 (6%)
Major bleeding	38 (4%)	210 (4%)	29 (5%)	151 (7%)	46 (6%)
Missing GDF-15	76 (9%)	365 (7%)	19 (3%)	171 (8%)	87 (12%)

Number in brackets represents number of missings.

AF, atrial fibrillation; BMI, body mass index; CKD-EPI, Chronic Kidney Disease Epidemiology Collaboration equation; GDF-15, growth differentiation factor 15; hs-cTnT, high-sensitive cardiac troponin T; NT-proBNP, N-terminal pro B-type natriuretic peptide; Q, quartile; RE-LY, Randomized Evaluation of Long-Term Anticoagulation Therapy; TIA, transient ischaemic attack.

### Association of GDF-15 with major bleeding by geographic region

**Figure 1:**
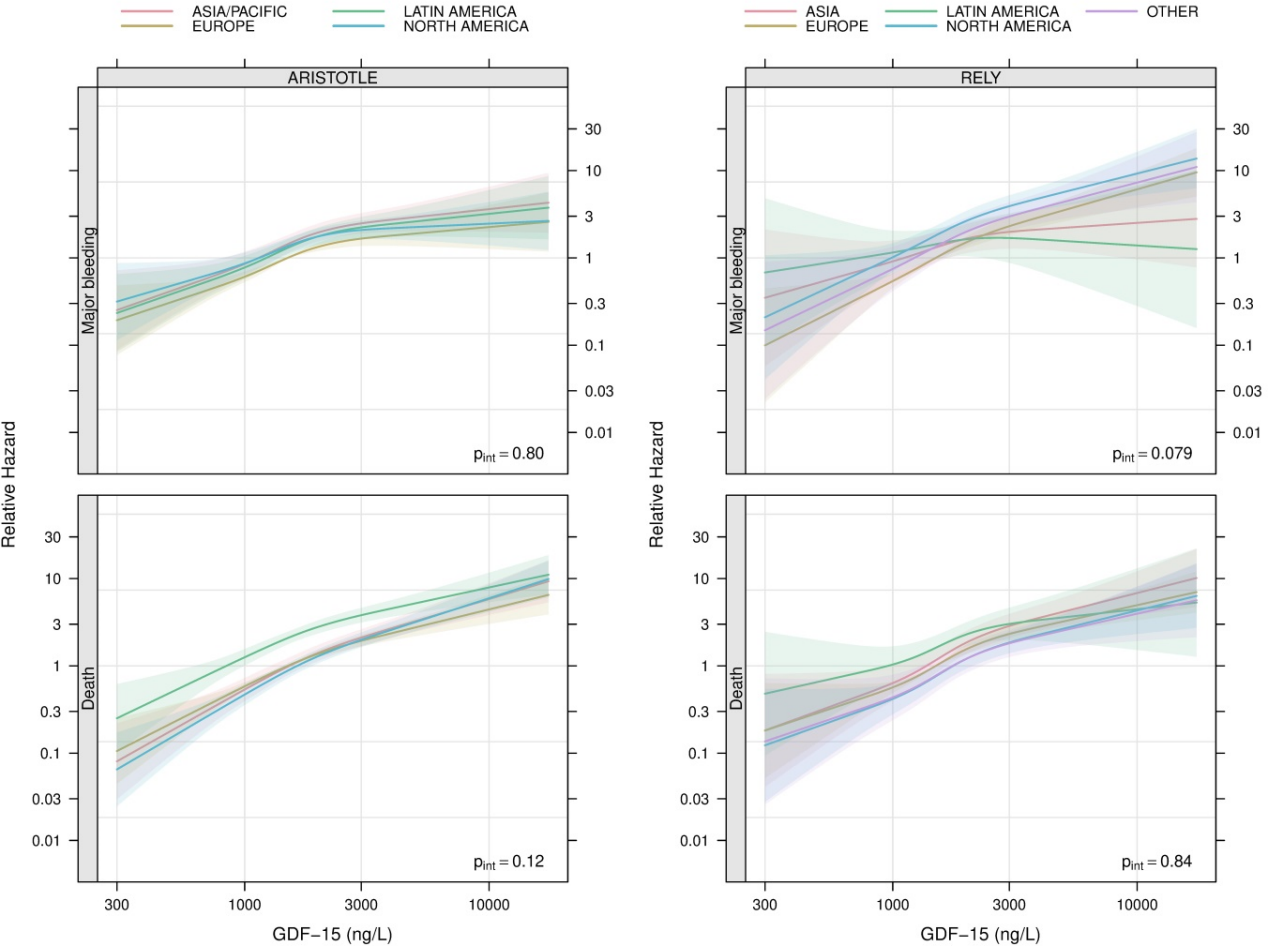
Relative hazard of major bleeding (top panels) and death (bottom panels) in relation to levels of GDF-15 among patients from different regions in the ARISTOTLE (left panels) and RE-LY (right panels) trials. An arbitrary reference point is set at a GDF-15 value of 1500 ng/L in Europe. The p value in each panel is for a test of no interaction between region and GDF-15. ARISTOTLE, Apixaban for Reduction in Stroke and Other Thromboembolic Events in Atrial Fibrillation; GDF-15, growth differentiation factor 15; RE-LY, Randomized Evaluation of Long-Term Anticoagulation Therapy.

In the ARISTOTLE biomarker cohort, GDF-15 was associated with major bleeding across all the study geographic regions (p<0.0001). The risk of major bleeding differed among regions (p=0.02) but there was no statistically significant interaction between GDF-15 and specific regions (p=0.80). The associations between GDF-15 and major bleeding by different regions are shown in figure 1 (top left panel).

In the RE-LY biomarker cohort, the results were similar, as GDF-15 was associated with major bleeding (p<0.0001). The risk of bleeding varied among regions (p<0.0001) as was the case in the ARISTOTLE biomarker cohort, the interaction between GDF-15 and specific regions was not statistically significant (p=0.08). The associations between GDF-15 and major bleeding by regions in the RE-LY biomarker cohort are shown in figure 1 (top right panel).

### Association of GDF-15 with death by geographic region

GDF-15 was associated with death in the ARISTOTLE biomarker cohort (p<0.0001). The risk of death differed among regions (p<0.0001) but the interaction between GDF-15 and regions was not statistically significant (p=0.12). The associations between GDF-15 with death by regions are shown in figure 1 (bottom left panel).

In the RE-LY biomarker cohort, the results were similar (figure 1, bottom right panels), with a strong association between GDF-15 and death (p<0.0001). Similarly, the mortality risk differed between regions (p=0.005) and the interaction between GDF-15 and regions was not statistically significant (p=0.84).

### Geographic evaluation of the ABC-AF-bleeding risk score

**Figure 2:**
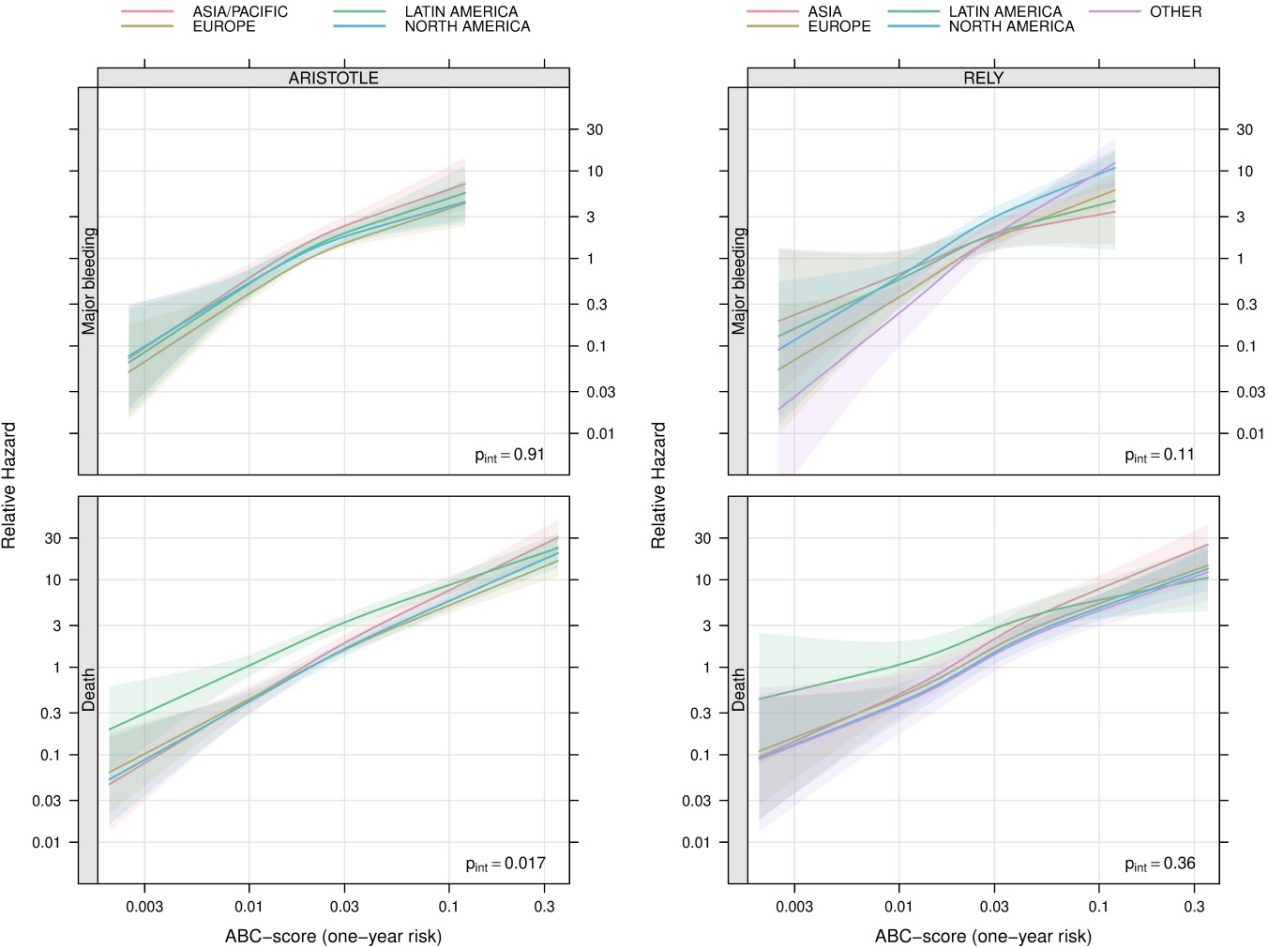
Relative hazard of major bleeding (top panels) and death (bottom panels) in relation to predicted ABC-AF-bleeding (top) and ABC-AF-death (bottom) 1-year risks among patients from different regions in the ARISTOTLE (left panels) and RE-LY (right panels) trials. An arbitrary reference point is set at a predicted ABC-AF-risk value of 0.02 in Europe. The p value in each panel is for a test of no interaction between region and predicted ABC-AF-risk. ABC-AF bleeding score is age, biomarkers (troponin-hs, haemoglobin, and GDF-15 or renal function), clinical history (previous bleeding). ARISTOTLE, Apixaban for Reduction in Stroke and Other Thromboembolic Events in Atrial Fibrillation; RE-LY, Randomized Evaluation of Long-Term Anticoagulation Therapy.

In the ARISTOTLE biomarker cohort, the ABC-AF-bleeding score was associated with major bleeding across all regions (p=0.0001) without a statistically significant interaction between the ABC-AF-bleeding score and regions (p=0.91). The associations between the ABC-AF-bleeding score with major bleeding by regions are shown in figure 2 (top left panel).

In the RE-LY biomarker cohort, the results were similar (figure 2, top right panel), as the ABC-AF-bleeding risk score was associated with major bleeding across geographic regions (p=0.0001) without a statistically significant interaction between the ABC-AF-bleeding score and regions (p=0.11).

Comparison of the discrimination of the ABC-AF-bleeding risk score showed consistency across all studied geographic regions in both the ARISTOTLE and RE-LY trial cohorts (table 3).

**Table 3:** C-indices with 95% CI for the ABC-AF-bleeding risk score in ARISTOTLE and RE-LY comparing geographic regions

	ARISTOTLE		RE-LY	
	C-index	95% CI	C-index	95% CI
Major bleeding				
All	0.677	0.657 to 0.698	0.708	0.684 to 0.731
Asia/Pacific	0.684	0.639 to 0.728	0.649	0.558 to 0.739
Europe	0.680	0.641 to 0.719	0.701	0.664 to 0.739
Latin America	0.674	0.629 to 0.719	0.657	0.561 to 0.754
North America	0.659	0.620 to 0.699	0.710	0.672 to 0.749
Other			0.760	0.698 to 0.822

ABC-AF bleeding score, Age, Biomarkers (troponin T-hs, haemoglobin, and GDF-15), Clinical history (previous bleeding); ARISTOTLE, Apixaban for Reduction in Stroke and Other Thromboembolic Events in Atrial Fibrillation; RE-LY, Randomized Evaluation of Long-Term Anticoagulation Therapy.

### Geographic evaluation of the ABC-AF-death risk score

In both the ARISTOTLE and RE-LY biomarker cohorts, the ABC-AF-death score was associated with all-cause mortality across all regions (p=0.0001) (figure 2, bottom panels). However, in both cohorts, we observed a higher mortality at lower ABC-AF-death scores in Latin America than in the other regions leading to a significant quantitative interaction with region in the ARISTOTLE cohort (interaction—p=0.018) although not in the RE-LY cohort (p=0.365) (figure 2, bottom panels).

The discrimination of the ABC-AF-death risk score was similar among geographic regions in both trial cohorts (table 4).

**Table 4:** C-indices with 95% CI for the ABC-AF-death risk score in ARISTOTLE and RE-LY comparing geographic regions.

	ARISTOTLE		RE-LY	
	C-index	95% CI	C-index	95% CI
Death				
All	0.743	0.728 to 0.759	0.741	0.721 to 0.761
Asia/Pacific	0.765	0.725 to 0.805	0.806	0.760 to 0.852
Europe	0.740	0.712 to 0.767	0.735	0.707 to 0.762
Latin America	0.715	0.686 to 0.743	0.677	0.606 to 0.749
North America	0.763	0.733 to 0.793	0.743	0.693 to 0.792
Other			0.734	0.662 to 0.806

ABC-AF death score, Age, Biomarkers (NT-proBNP, troponin T-hs and GDF-15), Clinical history (heart failure); ARISTOTLE, Apixaban for Reduction in Stroke and Other Thromboembolic Events in Atrial Fibrillation; RE-LY, Randomized Evaluation of Long-Term Anticoagulation Therapy.

## Discussion

The major findings of this study were that GDF-15 and the ABC-AF-bleeding and ABC-AF-death scores were consistently associated with major bleeding and mortality across all studied geographic regions. There was no significant interaction between GDF-15 in regard to outcomes by regions in either trial cohort. Results were similar for the ABC-AF-risk scores although there seemed to be a quantitative interaction between ABC-AF-death score and region with a higher mortality at lower ABC-AF-death scores in Latin America than other regions. The ABC-AF-bleeding and ABC-AF-death risk scores were consistent regarding their discriminative ability when comparing geographic regions in both the ARISTOTLE and the RE-LY trial cohorts.

Among the various biomarkers and risk scores that have been explored in regard to AF and its complications, none have to our knowledge been systematically examined regarding risk across geographic regions. Geographic differences could affect the predictive ability of these biomarkers and risk scores, making them less reliable in certain regions. This could be attributable to a number of factors, including genetic variation and regional treatment differences. When compared with other regions, for example, AF patients from Asia have both a higher risk of ischaemic stroke and bleeding.^10–13^ It is unknown how this increased risk in a specific region interacts with the risk associated with biomarkers and risk scores in AF. The assessment of geographic variation might therefore be helpful as part of a validation process when introducing a new biomarker or risk score intended for a wide use.

In the present study, there was a strong association between GDF-15, the ABC-AF-bleeding score and ABC-AF-death risk score with major bleeding and death independent of geographic regions and countries. The slope in figures 1 and 2 appears steeper for the ABC-AF- scores as compared with GDF-15 possibly implying a better discriminatory and risk assessment power of the ABC-AF- risk scores that most likely can be attributed the ABC-AF scores also including other predictive variables. There was a statistically significant interaction between the ABC-AF-death score and all-cause mortality for geographic regions in the ARISTOTLE cohort. This interaction could be explained by a somewhat weaker association between the ABC-AF-death score and death in Latin America where patients had a generally higher mortality risk as compared with the other regions, especially for those having a low ABC-AF-death score. Although the interaction was not statistically significant, a similar pattern was seen in the RE-LY cohort suggesting a possibly weaker association between the ABC-AF-death score and mortality in Latin America compared with the other regions. However, as the discriminatory ability of the ABC-AF-death score was consistent across geographic regions, this eventual interaction is likely to be less relevant from a clinical perspective. Otherwise, the variability between the regions concerning the association with the outcomes for GDF-15 and the ABC-AF scores, that is, the slopes of the lines in figures 1 and 2, were almost negligible in comparison with the strong associations with outcomes within the regions.

The findings from this study therefore indicate an overall consistency of the performance of GDF-15 and the ABC-AF-risk scores across geographic regions.

Although this is the first study to systematically explore geographic variation concerning risk association of GDF-15 and the ABC-AF-risk scores, some limitations and strengths may be worth mentioning. The results are based on post hoc analyses, and it is well known that lowering the number of events by creating subgroups reduces the statistical power to detect true differences. In order to increase the certainty of the results and to minimise the risk of random findings, we used data from two cohorts as any inconsistency appearing in both trials would lower the risk of it being a chance finding. Even though a large number of patients from a variety of countries were represented in each region, the results of these analyses are limited to the participating countries. Further, the large sample size including two different trial cohorts with a global patient representation of contemporary data, using similar variable definitions and outcomes, are some of the major strengths of this study. Despite convincing documentation, the ABC-AF scores currently are not routinely used. Part of that may be due to additional time and cost to obtain biomarker levels, but another component might have been the lack of specific validation across geographic areas which is now eliminated. The findings from this study thus add novel information regarding the consistency of GDF-15 and the ABC-AF-risk scores in AF and strengthen their role as a risk refinement tool applicable for a wide international usage.

## Conclusions

The associations between GDF-15 and the biomarker-based ABC-AF-bleeding and ABC-AF-death risk scores including GDF-15 and the outcomes major bleeding and death, in patients with AF on anticoagulation, are consistent across geographic regions. Accordingly, the discriminatory abilities of the ABC-AF-bleeding and ABC-AF-death scores to prognosticate major bleeding and death are consistent and similarly clinically useful across global geographic regions. The assessment of geographic variation might be helpful as part of future validation processes when introducing new biomarkers or risk scores intended for a wide use.

## Data Availability

The data underlying this article will be shared on reasonable request to the corresponding author.
