# Supplementary material for: Evaluation of the prognostic value of GDF-15, ABC-AF-bleeding score and ABC-AF-death score in patients with atrial fibrillation across different geographical areas
Source: Open Heart. 2021 Mar 19;8(1):e001471. doi: 10.1136/openhrt-2020-001471 (PMC7986788; doi:10.1136/openhrt-2020-001471)
Supplement: Supplementary data [file openhrt-2020-001471supp001.pdf]

Supplementary material

Evaluation of the prognostic value of GDF-15 and ABC-AF scores in different geographical areas

Tymon Pol, M.D., Ziad Hijazi, M.D., Ph.D., Johan Lindbäck, M.Sc., John H. Alexander, M.D., M.H.S., M. Cecilia Bahit, M.D., Raffaele De Caterina, M.D., Ph.D., John W. Eikelboom, M.D., Ph.D., Michael D. Ezekowitz M.D., Ph.D., Bernard J. Gersh, M.B, Ch.B., D.Phil., Christopher B. Granger, M.D., Elaine M. Hylek M.D., Ph.D., Renato D. Lopes, M.D., Ph.D., Agneta Siegbahn, M.D., Ph.D. and Lars Wallentin, M.D., Ph.D.

Contents

Supplementary Figure 1. 2

Supplementary Table 1. 3

Supplementary Table 2. 5

Supplementary Figure 1.

List of all included countries from both the ARISTOTLE and the RE-LY trial.

ARISTOTLE

ASIA/PACIFIC

Australia
China
Hong Kong
India
Japan
Malaysia
Philippines
Singapore
South Korea
Taiwan

EUROPE

Austria
Belgium
Czech
Denmark
Finland
France
Germany
Hungary
Israel
Italy
Netherlands
Norway
Poland
Romania
Russia
South Africa
Spain
Sweden
Turkey
UK
Ukraine

LATIN AMERICA

Argentina
Brazil
Chile
Colombia
Mexico
Peru
Puerto Rico

NORTH AMERICA

Canada
USA

RELY

ASIA

China
Hong Kong
India
Japan
Malaysia
Philippines
Singapore
South Korea
Taiwan
Thailand

EUROPE

Austria
Belgium
Bulgaria
Czech
Denmark
Finland
France
Germany
Greece
Hungary
Italy
Netherlands
Norway
Poland
Portugal
Romania
Russia
Slovakia
Spain
Sweden
Switzerland
Turkey
Ukraine
UK

LATIN AMERICA

Argentina
Brazil
Colombia
Mexico
Peru

NORTH AMERICA

Canada
USA

OTHER

Australia
Israel
South Africa

Pol T, *et al. Open Heart* 2022; 8:e001471. doi: 10.1136/openhrt-2020-001471

Supplementary Table 1. Baseline characteristics of the 14949 patients in the ARISTOTLE cohort according to study region.

	**ASIA/PACIFIC** *N* = 2389	**EUROPE** *N* = 6027	**LATIN AMERICA** *N* = 2959	**NORTH AMERICA** *N* = 3574
Age, Median (Q1 - Q3)	69.0 (61.0 - 75.0)	69.0 (62.0 - 75.0)	70.0 (64.0 - 77.0)	72.0 (65.0 - 78.0)
Female	35.7% (853)	37.1% (2235)	38.0% (1125)	31.0% (1109)
BMI, Median (Q1 - Q3)	25.1 (22.6 - 28.0) [7]	29.1 (26.1 - 32.8) [21]	28.7 (25.4 - 32.6) [24]	30.2 (26.5 - 35.0) [19]
Systolic blood pressure Median (Q1 - Q3)	130.0 (120.0 - 140.0) [8]	133.0 (123.0 - 143.0) [12]	130.0 (120.0 - 140.0) [2]	128.0 (117.0 - 138.0) [10]
Diabetes	24.7% (589)	22.7% (1370)	20.1% (595)	31.7% (1133)
Hypertension	81.0% (1936)	89.8% (5414)	88.8% (2629)	86.9% (3105)
Smoker	8.4% (200) [1]	9.9% (593) [9]	5.6% (166) [4]	7.2% (258) [0]
Permanent or persistent AF	89.6% (2141) [0]	80.8% (4867) [1]	91.5% (2706) [1]	83.2% (2973) [1]
Stroke/TIA	26.7% (637)	19.0% (1148)	15.3% (454)	15.8% (565)
Prior bleeding	15.8% (377)	12.1% (732)	13.2% (392)	26.1% (932)
Prior anemia	3.8% (90) [0]	4.9% (298) [3]	3.5% (104) [6]	14.4% (514) [2]
Congestive heart failure	24.9% (596)	40.8% (2458)	33.4% (989)	16.8% (601)

Pol T, *et al. Open Heart* 2022; 8:e001471. doi: 10.1136/openhrt-2020-001471

Myocardial infarction	8.2% (196) [0]	15.0% (906) [0]	9.5% (280) [1]	15.2% (542) [0]
Peripheral arterial disease	2.5% (59) [0]	5.3% (317) [0]	3.7% (109) [1]	6.8% (244) [0]
CKD-EPI (mL/min/1.73m2), Median (Q1 - Q3)	59.0 (47.2 - 72.8) [1]	56.5 (46.4 - 67.8) [7]	57.0 (45.5 - 70.5) [0]	52.4 (42.1 - 63.2) [0]
GDF-15 (ng/L), Median (Q1 - Q3)	1476.5 (1049.8 - 2167.5) [29]	1263.0 (919.5 - 1865.0) [88]	1362.0 (973.0 - 2012.0) [34]	1559.0 (1074.5 - 2297.5) [31]
Haemoglobin (g/dL), Median (Q1 - Q3)	14.1 (13.0 - 15.3) [13]	14.3 (13.3 - 15.3) [13]	14.4 (13.4 - 15.5) [26]	14.1 (13.0 - 15.0) [18]
NT-proBNP (ng/L), Median (Q1 - Q3)	725.0 (377.8 - 1220.2) [13]	695.0 (339.2 - 1244.0) [57]	751.0 (397.0 - 1325.0) [10]	706.0 (370.0 - 1217.8) [8]
hs-cTnI (ng/L), Median (Q1 - Q3)	5.1 (3.0 - 10.4) [26]	5.3 (3.3 - 9.7) [91]	6.6 (3.8 - 12.8) [16]	4.9 (2.9 - 8.7) [26]
hs-cTnT (ng/L), Median (Q1 - Q3)	9.8 (7.0 - 14.7) [12]	10.8 (7.4 - 16.3) [53]	11.4 (7.9 - 17.6) [9]	11.7 (7.9 - 17.6) [9]
**Outcomes**				
Death	152 (6%)	330 (5%)	332 (11%)	245 (7%)
Major bleeding	130 (5%)	206 (3%)	138 (5%)	195 (5%)
Missing GDF-15	28 (1%)	88 (1%)	34 (1%)	31 (1%)

AF indicates atrial fibrillation; BMI, body mass index; CKD-EPI, Chronic Kidney Disease Epidemiology Collaboration equation; Interleukin-6; Q, quartile; TIA, transient ischemic attack and number in brackets represents number of missings.

Pol T, *et al. Open Heart* 2022; 8:e001471. doi: 10.1136/openhrt-2020-001471

Supplementary Table 2. Baseline characteristics of the 9369 patients in the RE-LY cohort according to study region.

	**ASIA**<br>*N* = 891	**EUROPE**<br>*N* = 5024	**LATIN AMERICA**<br>*N* = 629	**NORTH AMERICA**<br>*N* = 2082	**OTHER**<br>*N* = 743
Age, Median (Q1 - Q3)	69.0 (63.0 - 75.0)	72.0 (66.0 - 77.0)	73.0 (67.0 - 78.0)	74.0 (68.0 - 79.0)	74.0 (69.0 - 79.0)
Female	32.9% (293)	36.9% (1855)	40.1% (252)	34.1% (711)	40.0% (297)
BMI, Median (Q1 - Q3)	24.3 (22.0 - 27.0) [3]	28.1 (25.4 - 31.4) [1]	27.9 (25.3 - 31.6) [0]	29.0 (25.7 - 33.3) [3]	28.4 (25.3 - 31.6) [0]
Systolic blood pressure, Median (Q1 - Q3)	130.0 (120.0 - 140.0) [2]	132.0 (120.0 - 145.0) [8]	131.0 (120.0 - 144.0) [0]	128.0 (118.0 - 140.0) [2]	130.0 (120.0 - 143.8) [1]
Diabetes	25.3% (225)	20.4% (1024)	16.2% (102)	24.4% (509)	29.5% (219)
Hypertension	71.6% (638)	78.7% (3954)	82.0% (516)	81.4% (1694)	79.3% (589)
Smoker	8.4% (75)	8.6% (432)	4.8% (30)	6.3% (132)	7.0% (52)
Permanent or persistent AF	69.7% (621) [0]	71.3% (3580) [0]	87.3% (549) [0]	55.2% (1147) [4]	58.7% (436) [0]
Stroke/TIA	29.4% (262)	18.8% (944)	14.9% (94)	16.8% (349)	23.4% (174)
Anemia	22.2% (198)	10.2% (513)	7.5% (47)	14.0% (291)	18.0% (134)
Congestive heart failure	34.9% (311) [0]	33.8% (1696) [0]	33.9% (213) [0]	16.0% (334) [1]	21.1% (157) [0]
Myocardial infarction	10.2% (91)	17.2% (864)	7.5% (47)	19.6% (408)	24.1% (179)

Pol T, *et al. Open Heart* 2022; 8:e001471. doi: 10.1136/openhrt-2020-001471

Peripheral arterial disease	0.4% (4) [0]	3.8% (193) [0]	2.2% (14) [0]	5.0% (105) [0]	3.9% (29) [1]
CKD-EPI (mL/min/1.73m2), Median (Q1 - Q3)	70.7 (58.5 - 85.4) [0]	65.5 (54.5 - 76.9) [75]	57.9 (47.5 - 68.3) [0]	64.2 (53.3 - 76.4) [8]	63.1 (52.2 - 75.2) [8]
GDF-15 (ng/L), Median (Q1 - Q3)	1606.0 (1181.5 - 2407.5) [76]	1436.0 (1060.0 - 2067.0) [365]	1545.0 (1142.2 - 2081.2) [19]	1608.0 (1182.0 - 2332.0) [171]	1744.5 (1231.5 - 2605.8) [87]
Hemoglobin (g/dL), Median (Q1 - Q3)	13.9 (12.7 - 15.1) [3]	14.4 (13.4 - 15.4) [103]	14.4 (13.4 - 15.5) [4]	14.1 (13.1 - 15.1) [29]	14.0 (12.8 - 15.0) [20]
NT-proBNP (ng/L), Median (Q1 - Q3)	807.0 (400.5 - 1576.5) [3]	837.0 (396.0 - 1477.0) [35]	819.0 (453.0 - 1410.0) [8]	748.0 (368.5 - 1354.0) [19]	854.0 (372.5 - 1406.5) [8]
hs-cTnI (ng/L), Median (Q1 - Q3)	6.8 (4.2 - 14.8) [33]	7.2 (4.7 - 13.0) [290]	7.3 (4.4 - 14.0) [18]	5.6 (3.5 - 10.0) [102]	7.4 (4.5 - 15.0) [11]
hs-cTnT (ng/L), Median (Q1 - Q3)	10.8 (7.1 - 18.0) [39]	12.2 (7.7 - 19.5) [385]	12.9 (8.3 - 20.0) [21]	12.0 (7.6 - 19.0) [121]	13.8 (8.5 - 22.8) [84]
**Outcomes**					
Death	66 (7%)	341 (7%)	54 (9%)	104 (5%)	42 (6%)
Major bleeding	38 (4%)	210 (4%)	29 (5%)	151 (7%)	46 (6%)
Missing GDF-15	76 (9%)	365 (7%)	19 (3%)	171 (8%)	87 (12%)

AF indicates atrial fibrillation; BMI, body mass index; CKD-EPI, Chronic Kidney Disease Epidemiology Collaboration equation; Q, quartile; TIA, transient ischemic attack and number in brackets represents number of missings.
